# How the pilidium larva feeds

**DOI:** 10.1186/1742-9994-10-47

**Published:** 2013-08-09

**Authors:** George von Dassow, Richard B Emlet, Svetlana A Maslakova

**Affiliations:** 1 Oregon Institute of Marine Biology, University of Oregon, Charleston, OR, USA

**Keywords:** Nemertea, Pilidiophora, Planktotrophy, Larval ecology, Ciliary particle capture

## Abstract

**Introduction:**

The nemertean pilidium is a long-lived feeding larva unique to the life cycle of a single monophyletic group, the Pilidiophora, which is characterized by this innovation. That the pilidium feeds on small planktonic unicells seems clear; how it does so is unknown and not readily inferred, because it shares little morphological similarity with other planktotrophic larvae.

**Results:**

Using high-speed video of trapped lab-reared pilidia of *Micrura alaskensis*, we documented a multi-stage feeding mechanism. First, the external ciliation of the pilidium creates a swimming and feeding current which carries suspended prey past the primary ciliated band spanning the posterior margins of the larval body. Next, the larva detects prey that pass within reach, then conducts rapid and coordinated deformations of the larval body to re-direct passing cells and surrounding water into a vestibular space between the lappets, isolated from external currents but not quite inside the larva. Once a prey cell is thus captured, internal ciliary bands arranged within this vestibule prevent prey escape. Finally, captured cells are transported by currents within a buccal funnel toward the stomach entrance. Remarkably, we observed that the prey of choice – various cultured cryptomonads – attempt to escape their fate.

**Conclusions:**

The feeding mechanism deployed by the pilidium larva coordinates local control of cilia-driven water transport with sensorimotor behavior, in a manner clearly distinct from any other well-studied larval feeding mechanisms. We hypothesize that the pilidium’s feeding strategy may be adapted to counter escape responses such as those deployed by cryptomonads, and speculate that similar needs may underlie convergences among disparate planktotrophic larval forms.

## Introduction

Among the zooplankton live numerous animal larvae that feed on small phytoplankton. The feeding mechanisms for three archetypical forms are relatively well understood: nauplii and the nauplius-like copepod adults use muscular movements of cuticularized combs to collect and transport food mouthward [1,2]; dipleurulas of echinoderms and hemichordates use local ciliary reversals to direct desirable cells toward the mouth against the prevailing ciliary current [3-5]; and most trochophore-type larvae among the spiralians use two closely-apposed oppositely-directed bands of compound cilia to move particles into a ciliated groove between the bands in which the current runs toward the mouth [6-8]. There exist numerous variations on these themes, such as adaptations to capture large food (e.g. [9,10]), as well as novelties such as the hood-flapping suction behavior of phoronid actinotrochs [11,12], and there are several larval forms for which the mechanism of food gathering remains unclear.

The pilidium is the characteristic larva of the monophyletic nemertean clade Pilidiophora, including the order Heteronemertea plus the palaeonemertean genus Hubrechtella [13,14]. Like so many long-lived planktotrophic larvae, the pilidium consists of a thin epithelium stretched over a vast fluid cavity and divided into zones by an actively-beating ciliated band. In the conventional pilidium, such as the original type (pilidium gyrans) described by Johannes Müller [15], the primary ciliated band is elaborated into lobes and lappets which, because of the direction of ciliary beat, trail the larva as it swims apical tuft first. The overall resemblance to a hat with ear flaps is reflected in the very name of the larva (“pileos” is Greek for “cap”). Between the lappets is a funnel-shaped esophagus or buccal cavity, at the top of which is the entrance to a small, round stomach; which entrance is also the exit. As the pilidium feeds and grows, small patches of tissue – imaginal discs – develop in defined locations within the larval body, and then fuse to form the juvenile body. When complete, the juvenile escapes its larval vehicle and takes up a benthic existence, devouring the larval tissues as its first meal [16-18].

Nothing quite like the pilidium appears outside of this one clade of nemerteans. The sister clade Hoplonemertea is characterized by lecithotrophic planuliform larvae termed “decidulas” because they possess, and then shed or resorb, a transitory larval epidermis [19-21]. Although the internal development of decidula-type hoplonemertean larvae may yet turn out to have some similarities to the pilidium [21], feeding by the decidulas has not been described. Among the basal assemblage of palaeonemerteans, all known larvae are elongated planuliform creatures which, if they feed at all, feed on large prey ([22,23], Maslakova pers. obs.); in one group the larva has been shown to possess a cryptic prototroch [24], but there is no evidence of a ciliary mechanism for food capture. In other phyla, the Müller's larva of turbellarian flatworms and the cyphonautes of bryozoans have superficial similarity to the pilidium, mostly in the configuration and innervation of ciliated bands relative to the mouth and the direction of swimming [25,26], but no obvious homologies. Therefore it presently appears that the pilidium is a novel invention of one group of nemerteans, and that whatever mechanism by which it feeds must also have been invented more or less anew.

We describe here, from studies of high-speed video microscopy, how the nemertean pilidium feeds on small flagellated unicells. Although the posture of the lappets is such that the larva does juxtapose two ciliated bands that beat toward one another, as in opposed-band feeding of trochophores and molluscan veligers, the capture mechanism in no way relies on this configuration. Instead, local control of rapid muscular deformations of the lobes and lappets direct parcels of food-containing water into the buccal funnel, where secondary ciliated bands apparently function to retain captured cells while tractoring them slowly toward the stomach entrance and simultaneously expelling water. As far as we are aware the pilidial feeding mechanism is unique, though it has clear parallels to other particle capture devices as diverse as the phoronid actinotroch, the Venus' flytrap, a fluorescence-activated cell sorter, and baleen whales.

## Results

### Overview of pilidial anatomy

**Figure 1:**
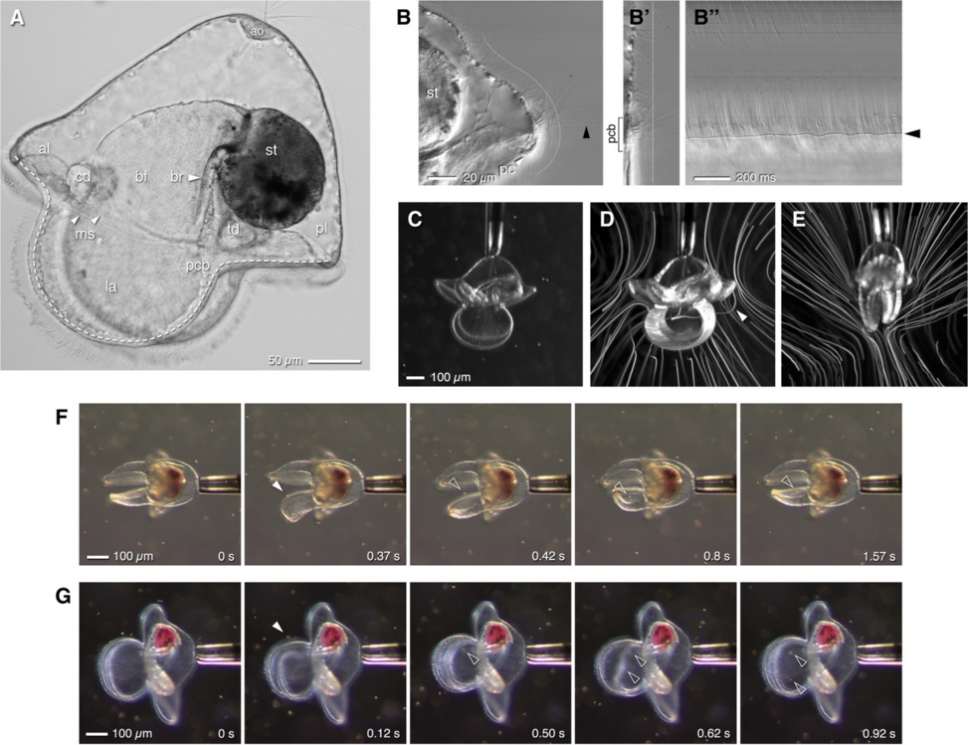
**The pilidium snatches food from its flow field.** All images show pilidia of Micrura alaskensis. **(A)** ~2-wk-old pilidium illustrating larval anatomy: apical organ (ao); anterior and posterior lobes (al, pl); lappets (la); primary ciliated band (pcb, dashed line); buccal funnel (bf); buccal ridge (br); stomach (st); cephalic disc (cd); trunk disc (td); muscles (ms; upper fiber is the largest circumesophageal muscle, lower one flattens lappets together). **(B)** Mid-saggital plane, posterior lobe, contrasting ciliation of larval epidermis and primary ciliated band (frame from Video 1). The line indicated, starting from the posterior cirrus (pc) and running halfway up the midline toward the apical organ, was straightened **(B)**’ to make a kymograph **(B)**”, wherein the non-beating cilium is apparent (arrowhead). Note the difference in beat between epidermal cilia (top of kymograph) and the primary ciliated band: slope and sweep are both greater in the primary ciliated band. The empty zone in the middle of the kymograph reflects that the epidermal cilia immediately apical of the band are shorter than elsewhere. **(C)** 4-week-old lab-raised pilidium at advanced proboscis stage, held by capillary and surrounded by Rhodomonas (bright dots); high-speed video of this larva was brightest-point projected to make panel **D**. **(D)** Typical flow field from the side. Note the path of a captured cell (arrowhead) which rides over posterior lobe, then between lappets. **(E)** Flow field around a similar larva as **C**, held in frontal view (i.e., looking along the slot between the lappets). **(F,G)** Sequences from real-time video (Videos 2 and 3) showing frontal and side views of capture events. In F the larva flexes one lappet, gulping a single cell. In **G** the larva captures two cells in quick succession. Arrowheads indicate captured cells.

The embryos of a small sand-flat-dwelling heteronemertean *Micrura alaskensis* can be reared to metamorphosis within five to six weeks on a unialgal diet of the cryptomonad commonly known as *Rhodomonas lens*. Maslakova [18] provided a detailed description and timeline of larval development in this species; we briefly summarize relevant features of development and pilidial anatomy (Figure 1A) for orientation. Within one week of lab culture, pilidium larvae of *M. alaskensis* are helmet-shaped with a prominent apical tuft; they possess a pair of well-developed lateral lappets actively flexed by muscles; distinct anterior and posterior lobes (directions are relative to the body axis of the juvenile that will eventually develop inside the larval body); a deep buccal funnel leading to a round stomach; and, under favorable conditions, the developing anterior-most pair of imaginal discs (the cephalic rudiments). By two weeks (the larva shown in Figure 1A) the next pair of discs, the trunk rudiments, appear beneath the stomach. The larva propels itself through the water, apical tuft first, using a primary ciliary band that runs almost continually along the margins of the anterior and posterior lobes and the two lateral lappets (Figure 1A).

A prominent circumferential muscle demarcates the buccal funnel from the space between the lappets. This boundary, associated with an internal ridge described as “lips” in pilidia of other species by Coe (ref. [27] p.247), is often partly constricted, suggesting a functional division; to the extent that a pilidium has a mouth, this may be it. We therefore refer to the space between the lappets as the “vestibule” and reserve “buccal funnel” for the portion that is apical to the outermost constriction. The inner margin of each lateral lappet features an inner ciliated band, running parallel to the primary ciliated band. The other important part of the ciliary apparatus is a pair of densely ciliated buccal ridges running along the posterior wall of the buccal funnel from the posterior margin of the lateral lappets to the stomach entrance.

By three weeks the trunk discs become prominent and the third pair of imaginal discs (cerebral organ rudiments) invaginate from the inner epithelium of the lappet. By about four weeks the growth of juvenile rudiments fuses them into a toroid of imaginal tissue encircling the buccal funnel; thereafter, the extension of the proboscis and growth of a dorsal rudiment close over the top, until the stomach is entirely enclosed by the developing juvenile. The studies described here involved larvae between two and four weeks old. During this time, larvae grow but the relative proportions of larval parts change little, with the notable exception that the anterior and posterior lobes enlarge, to the point that the larva, seen from the top while swimming vigorously, looks a bit like a ship's propeller. Most cells of the larval epidermis are heavily multi-ciliated: throughout most of the epidermis the cilia are shorter (15-20 μm) and much less dense than the cilia in the primary ciliated band itself (~40 μm), but beat in the same direction (Figure 1B; Additional file 1: Video 1). Single long (>40 μm), non-beating cilia occur at regular intervals within the primary ciliated band (arrowhead in Figure 1B).

### Stream redirection by muscular flexion of the lobes and lappets

Pilidia of *M*. *alaskensis* feed avidly on dense suspensions of *Rhodomonas* and other cryptomonads, but not nearly so readily when restrained by compression between a slide and coverslip. We therefore used fire-polished capillary pipettes with 30-50 micron openings to hold larvae by suction (Figure 1C). Happily, two- to four-week-old pilidia seem largely insensitive to being held by the apical tuft, and feed eagerly while thus tethered (Figure 1F,G; Additional file 2: Video 2, Additional file 3: Video 3), although the capillary suction is often sufficient to draw in and deform the epidermis around the apical organ. Even then, we observed that suction-tethered pilidia recovered rapidly and completely after release. Suction-tethering enabled us to hold larvae in a swimming and feeding configuration, one to several millimeters from either glass or air-water interface. In this configuration we expect the motion of surrounding particles to approximate the relative motion of a freely-swimming larva through a particle suspension. Flowfields displayed by overlaying video frames (Figure 1D,E) illustrate how currents generated by the primary ciliated band draw cells in from a large sampled volume, and that this flow converges to the edge of the lappets; a narrow stream emerges from the margins of the lappets when they are relaxed and held together. Because larvae are held stationary in a fixed volume, cells are entrained from a broader region upstream (more concave trajectories) than if larvae were translating through the fluid; whether tethered or swimming, stream lines are compressed where cilia generate flow.

**Figure 2:**
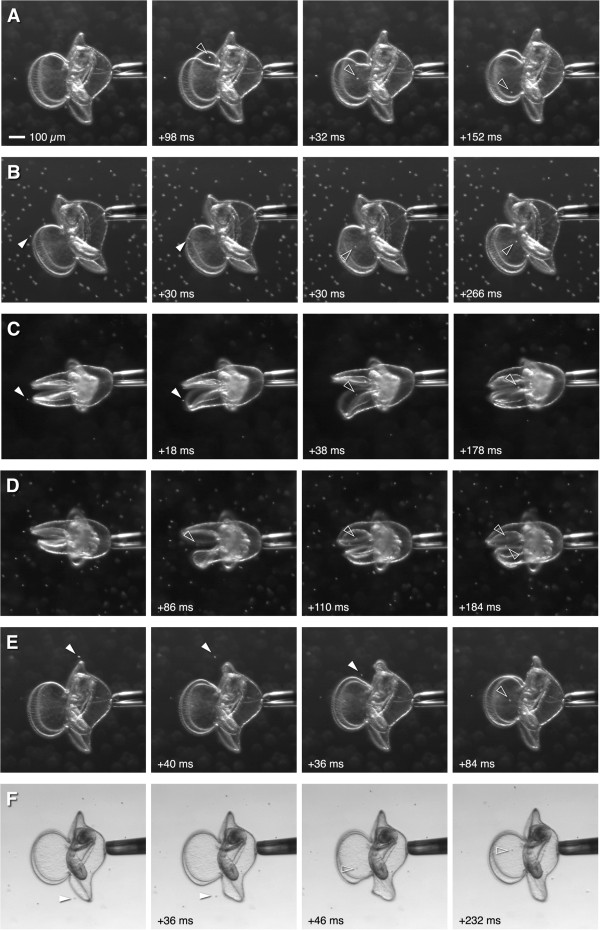
**The pilidium can independently deform any part of the ciliated band to redirect streams containing prey.** Frame sequences **(A-F)** correspond to the six high-speed video sequences assembled in Additional file 4: Video 4. Times indicate the change from the previous frame in the row. Arrowheads indicate the captured cells (solid arrowheads – before capture, open – after capture, i.e. passing into the vestibule). This set of recordings summarizes the pilidium’s characteristic repertoire of capture behaviors ranging from discrete sips to large gulps or multi-part captures. All of these larvae are four-week-old lab-reared *M. alaskensis*.

In real-time recordings taken at low magnification through a stereomicroscope, it became obvious that particle capture was associated with rapid flicks and twitches of the lappets and lobes. Larvae seemed to snap at cells, or gulp parcels of water that contain them (Figure 1F,G; Additional file 2: Video 2, Additional file 3: Video 3). The relation between these movements became clear, however, only from high-speed video at 500 fps or higher. These revealed that the flicks and twitches represent surprisingly local deformations of the lobes and lappets, seemingly timed and positioned to transiently re-direct a stream of food-containing water into the vestibule (Figure 2; Additional file 4: Video 4). The lappets – either one alone or both together – were observed to flex open along a portion of their circumference corresponding to a third or fourth of their perimeter. The lobes, in contrast, were observed to flex mouthward (i.e., toward the space between the lappets); in such instances the lappet edges adjacent to the flexing lobe also flexed open, at the same instant or just slightly afterward, to admit the food. These motions amounted to discrete ‘sips’ due to localized flexure of the lappets (Figure 2A,B), gulps in which a large parcel of water was sucked between the lappets (Figure 2C,D), or combinations involving both stream re-direction and suction (Figure 2E,F). Notably, the same individual larva (e.g. Figure 2A,E) executed diverse maneuvers within the course of a recording. During these initial captures, the engulfed cell typically transited from the lappet margin to center within 20-50 ms, a distance of ~100 μm, meaning that the pilidium moves its prey at 2-5 mm/s.

**Figure 3:**
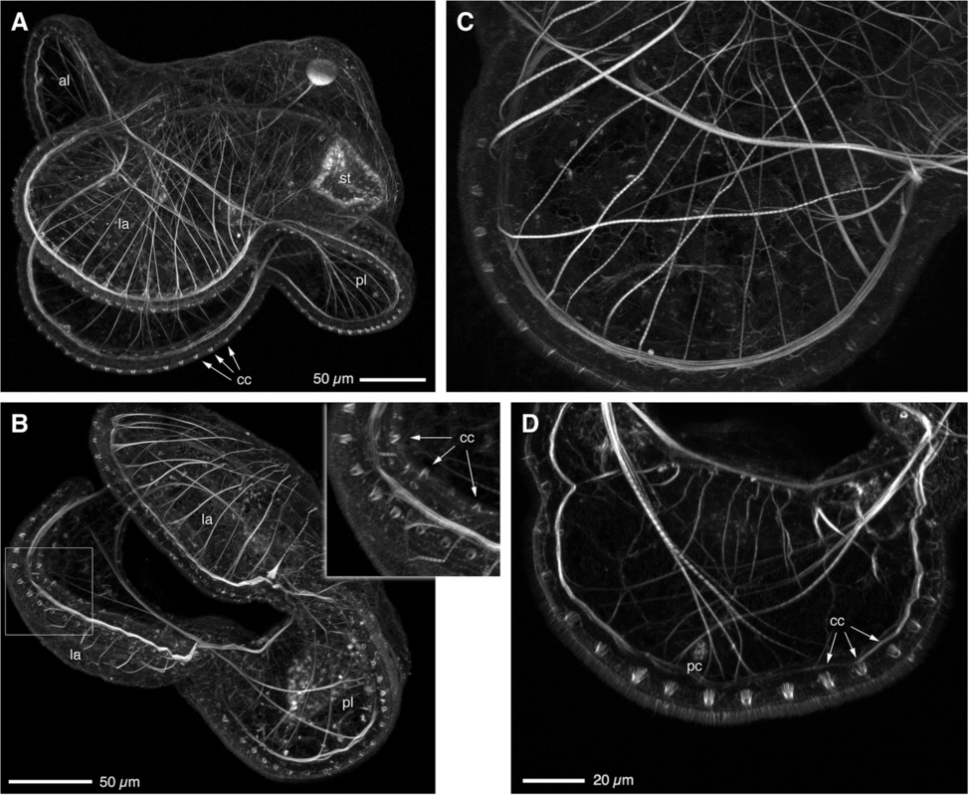
**A network of smooth and striated muscles operates the pilidial body.** All panels show brightest-point projections of confocal Z-series of *M*. *alaskensis* larvae fixed and stained with phalloidin. **(A)** Lateral view of ~4-week-old pilidium. Prominent muscle fibers run along the margins of anterior and posterior lobes (al, pl) and lappets (la), and also span the bases of lappets; radial muscle fibers running along the inner surface of the outer epithelium make connections to the lappet and lobe margins every 10-20 μm. Also apparent is the regular row of collar cells (cc) along the primary ciliated band. This panel was re-rendered from the same dataset as Ref. 21, Figure 5A. **(B)** View from underneath the posterior lobe of a 17-day-old pilidium, showing a similar musculature as in **(A)**, but sparser. The radial muscles of the lappet are striated. Note the bifurcation of radial muscles near the margin. Also note that the inner ciliated band in the lappets is also populated by collar cells (inset, arrows, cc). **(C)** Close-up of the lappet musculature in a 17-day-old pilidium. Most of the radial fibers are clearly striated; the marginal band is not. **(D)** Close-up of the musculature of the posterior lobe in a 17-day-old pilidium, illustrating that some of the radial fibers are striated, some are not (yet). Note also the posterior cirrus (pc) and the single row of collar cells (arrows, cc) in the primary ciliated band.

The pilidium can accomplish such rapid, local flexures of the larval margin presumably because it contains an elaborate musculature consisting of both smooth and striated muscle fibers (Figure 3A,B). Within the lappets, a bundle of smooth-staining fibers runs around the entire margin, immediately beneath the ciliated band, while striated fibers run almost radially, connecting to the periphery near the smooth bundle (Figure 3C). It seems likely that the smooth peripheral bundle is responsible for postural adjustments of the lappets, whereas the radial striated fibers are the obvious candidates to generate the flicks and flaps that capture food. The anterior and posterior lobes have similar arrangements of both peripheral smooth fibers and quasi-radial striated fibers (Figure 3D). Striations become more prominent as larvae grow (compare the posterior lobes in Figure 3B and D); curiously, it often appears that one region of a single fiber is striated and another region smooth (as in Figure 3D).

### Putative sensory cells embedded in the ciliated bands

**Figure 4:**
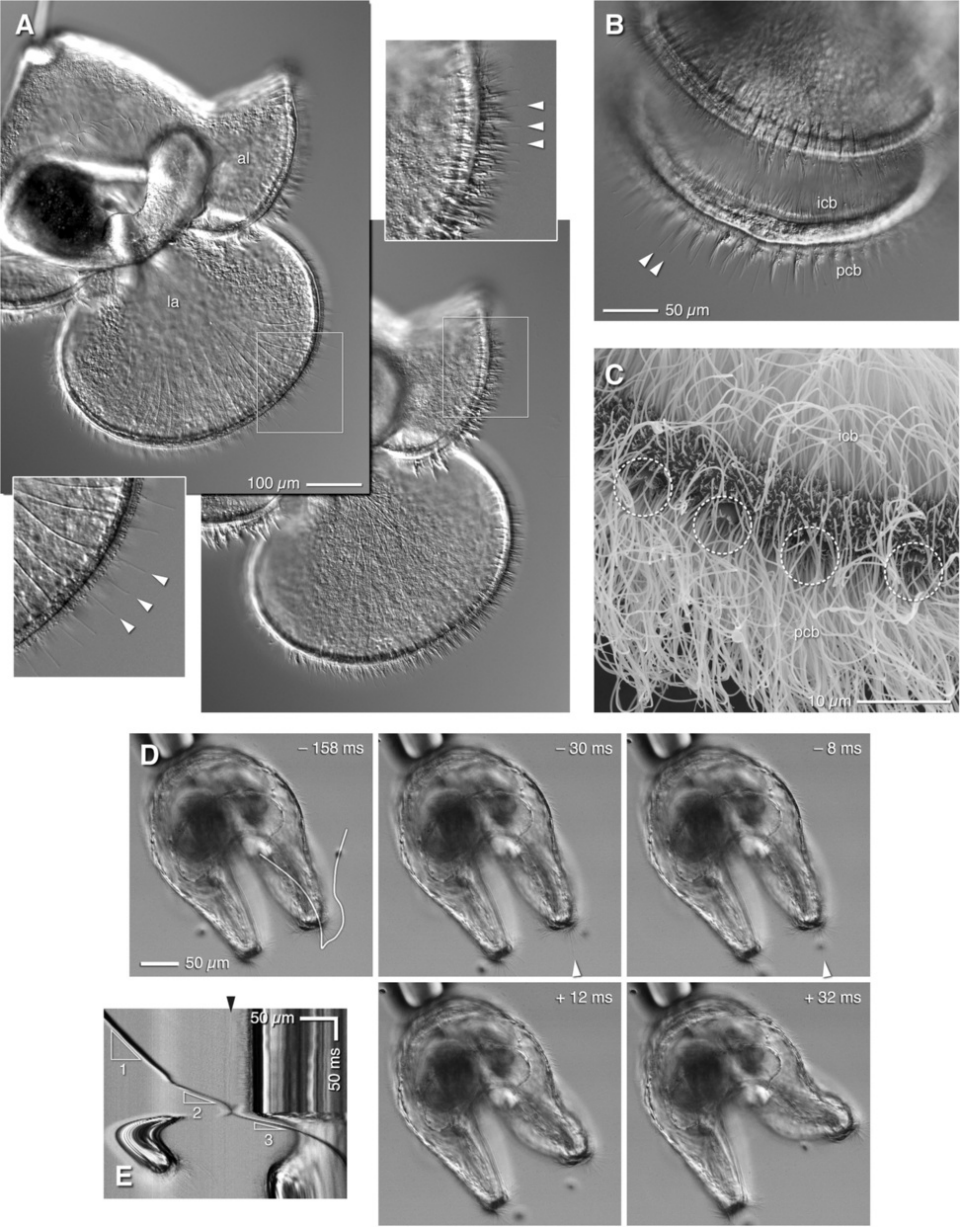
**Non-beating cilia are the candidate sensors that trigger particle capture. (A)** DIC micrographs of live, wild-caught advanced-proboscis-stage pilidium (likely of *Lineus flavescens* or a close relative thereof) at two different focal planes to show the non-beating cilia (arrowheads) and their posture in the inner ciliated band of the lappets (la) and the single ciliated band of the anterior lobe (al). Insets double the magnification for inner band of the lappet (bottom left) and anterior lobe (top right). **(B)** Unconstrained pilidium (same kind, developmental stage, and plankton sample as panel A) viewed askew into the lappets, showing the stationary cilia (arrowheads) amidst the metachronal wave of the primary ciliated band (pcb). Note also that the inner ciliated band (icb) lies nearly flat against the inside wall of the lappet. **(C)** Equivalent view of the lappet margin by SEM; four circles identify anemone-shaped uniciliate collar cells, which correspond to the non-beating cilia on panels A and B, and to the phalloidin-stained collars shown in Figure 3. **(D)** Video sequence of capture in which a cell passes the lappet margin nearly in focus (see Additional file 5: Video 5). The larva is in frontal view. Times are relative to the first frame in which the lappet moves. A non-beating cilium (arrowhead) is in focus. The cell to be captured pauses just beyond the position of this non-beating cilium immediately before the lappet moves to suck the cell in. **(E)** Kymograph of the straightened path indicated in the first frame of **(D)**. This depiction provides a readout of the captured cell’s speed, as indicated by triangles: 1 = 0.63 mm/s; 2 = 1.7 mm/s; 3 = 2.9 mm/s. A non-beating cilium is indicated by the black arrowhead, and the cell exhibits a pause of ~20 ms as it passes this point. Note that this cell is as much as 10 μm out of focus, and thus surely interacts with a different non-beating cilium than the one visible.

The vast majority of algal cells that we saw engulfed as a consequence of lappet or lobe flicks by pilidium larvae approached within a very short distance to the ciliated band; exceptional cases include instances in which, at very high food density, cells simply wandered between the lappets of larvae trapped between slide and coverslip. Among the beating cilia of the band there appear regularly-spaced non-beating cilia, slightly longer than the rest (Figure 4A,B; also Figure 1B). These appear to emerge from uniciliate cells that bear a microvillar collar around the base of the cilium; the collar is evident in phalloidin-stained larvae (collar cells in Figure 3) and by SEM (Figure 4C), and such cells are present in all of the pilidium's ciliated bands (see below for description of the various ciliary bands). In the primary ciliated band, both in the lobes and lappets, the collar cells are spaced 8-10 μm apart, which is strikingly similar to the diameter of the small flagellates pilidia accept as food (in the lappets’ inner band the spacing between collar cells is approximately doubled; Figure 4A). High-speed video sequences showed that the pilidium initiates a movement of its lappet within a few milliseconds of apparent contact between a prey item and one of the non-beating cilia associated with the primary ciliated band (Figure 4D; Additional file 5: Video 5). The path of the cell to be captured (Figure 4D) exhibited a brief pause before the lappet moved to initiate engulfment (Figure 4E), and this pause took place at or just beyond the row of non-beating cilia associated with the primary ciliated band. This scenario occurred in at least eight recordings in which the captured cell passed near enough to the focal plane, and in which optical conditions made visible the non-beating cilia. We therefore surmise that these non-beating cilia are the sense organs which detect food items and trigger capture behaviors.

### The secondary ciliated band inside the lappets

**Figure 5:**
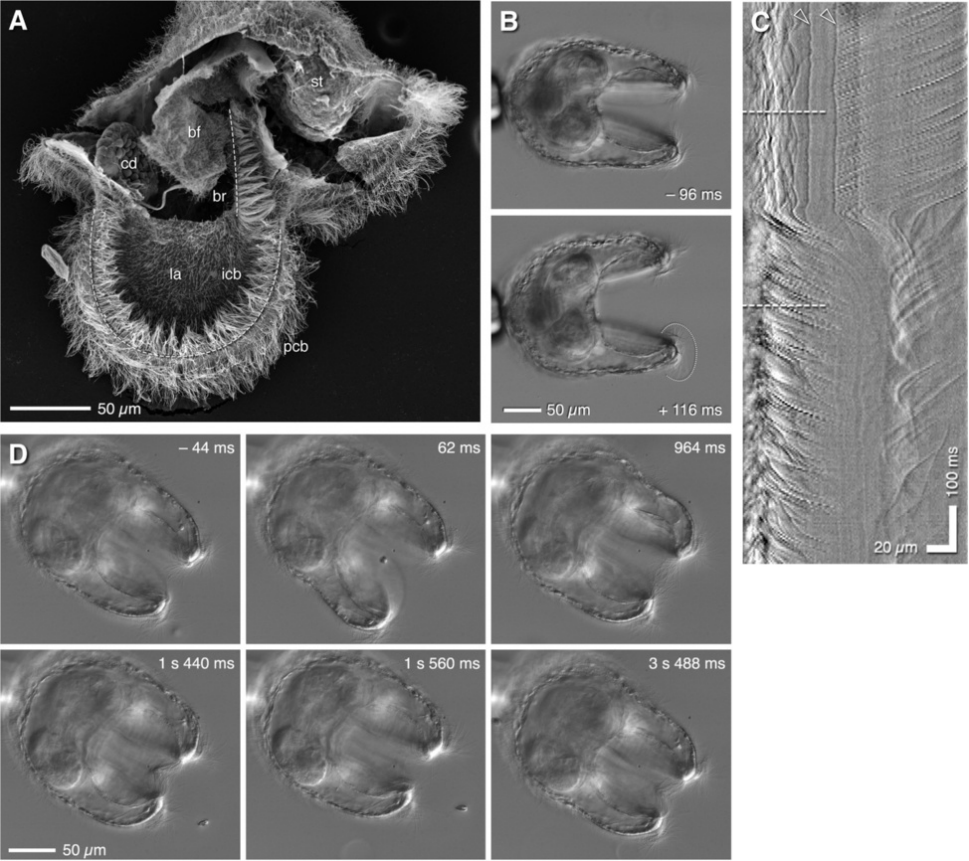
**The inner ciliated band beats outward after prey capture. (A)** SEM of fractured pilidium at the two-pairs-of-discs stage, exposing inner surface of one lappet (la) and buccal funnel (bf). Just inside the primary ciliated band (pcb), the inner ciliated band (icb) lies flat in most fixed larvae. This band is distinguished from the buccal ridge (br) by the offset at the margin between the lappet and the buccal funnel (dashed lines run along the bases of cilia in each band). cd = cephalic disc; st = stomach. **(B)** Two frames from Additional file 6: Video 6, before and after capture (times relative to lappet movement); larva in frontal view. Here the upper lappet moves as a cell is captured far out of focus. Both lappets activate the inner ciliated band and pause the primary band, as indicated by the recovery strokes. **(C)** Kymograph made by straightening the dotted line from the second frame of panel B. Traces show speed and beat orientation of ciliary tips. Dashed lines indicate times of frames of panel B. In the upper portion, the inner band (left side) shows no directed beat – traces meander like strands of wet hair – while the primary ciliary band beats strongly; the traces switch within ~15 ms near the middle of the sequence, such that the inner band beats out while the primary band stalls. Arrowheads indicate traces of two non-beating cilia, one within the inner band and one within the primary band, at the top of the kymograph. **(D)** Frames from a capture sequence (Additional file 7: Video 7) featuring a sustained barricade made by the inner ciliated bands. Times are relative to motion of the lappets. This pilidium transiently drops the barricade (frame 5), perhaps in response to another prey item passing within reach.

The primary ciliated band is the most prominent because it runs along the larval margin and beats consistently and vigorously. Inside the lappets (but not the anterior and posterior lobes) runs a secondary, inner ciliated band, separated from the primary band by a gap of ~5 μm. The inner ciliated band was described by Salensky [28] from histological sections of wild-caught pilidia from the Mediterranean, and described and illustrated with SEM by Nielsen [29] in wild-caught pilidia from Vietnam and Sweden. We have noted its presence in lab-reared pilidia of many species in the NE Pacific, and it is probably safe to say that it is present in all conventional (hat-like) pilidia. The inner band does not beat most of the time, and in fixed larvae these cilia are usually flattened against the inner surface of the lappet (Figure 5A; see also Figure 4B). We found that the inner band was activated immediately following food capture, and that the effective stroke of cilia in the inner band is away from the buccal funnel, toward the outer ciliated band (Figure 5B; Additional file 6: Video 6). Meanwhile, as the inner band began to beat, the beating of the primary ciliated band usually diminished or ceased entirely; this switch took place within milliseconds (Figure 5C) and persisted for as much as a second before the beat of the primary band was restored. Even after the primary ciliary band resumed beating, the inner cilia remained elevated. Because of the juxtaposition of the two lappets, the elevated cilia of the inner bands mesh across the gap such that together they appear to erect a fence across the gap between the lappets (Figure 5D; Additional file 7: Video 7). This barricade was maintained for several seconds.

We noted, however, that the barricade does not span the entire lappet perimeter: the inner band usually did *not* beat in the specific zone where the captured cell entered (Additional file 8: Video 8; also Figure 5D, frame 2, lower lappet), and in this zone the primary ciliated band continued to beat. Because recordings are constrained to a single focal plane and lappet movements typically far exceeded the depth of field, we could not discern over what fraction of the perimeter the ciliary beat changed. However, since we saw the inner band remain at rest only when the particle entered in or near focus, and since we observed the inner band erected rapidly when particles entered far from the focal plane, we suspect that the switch in beat happens over most of the lappet excepting a small zone around the entry site. From this we interpret that upon detecting a capture event, the lappets lower an entrance ramp in the capture zone; elsewhere, the outward beat of the inner band expels water while barricading the exits.

### Ciliated ridges within the esophageal funnel

**Figure 6:**
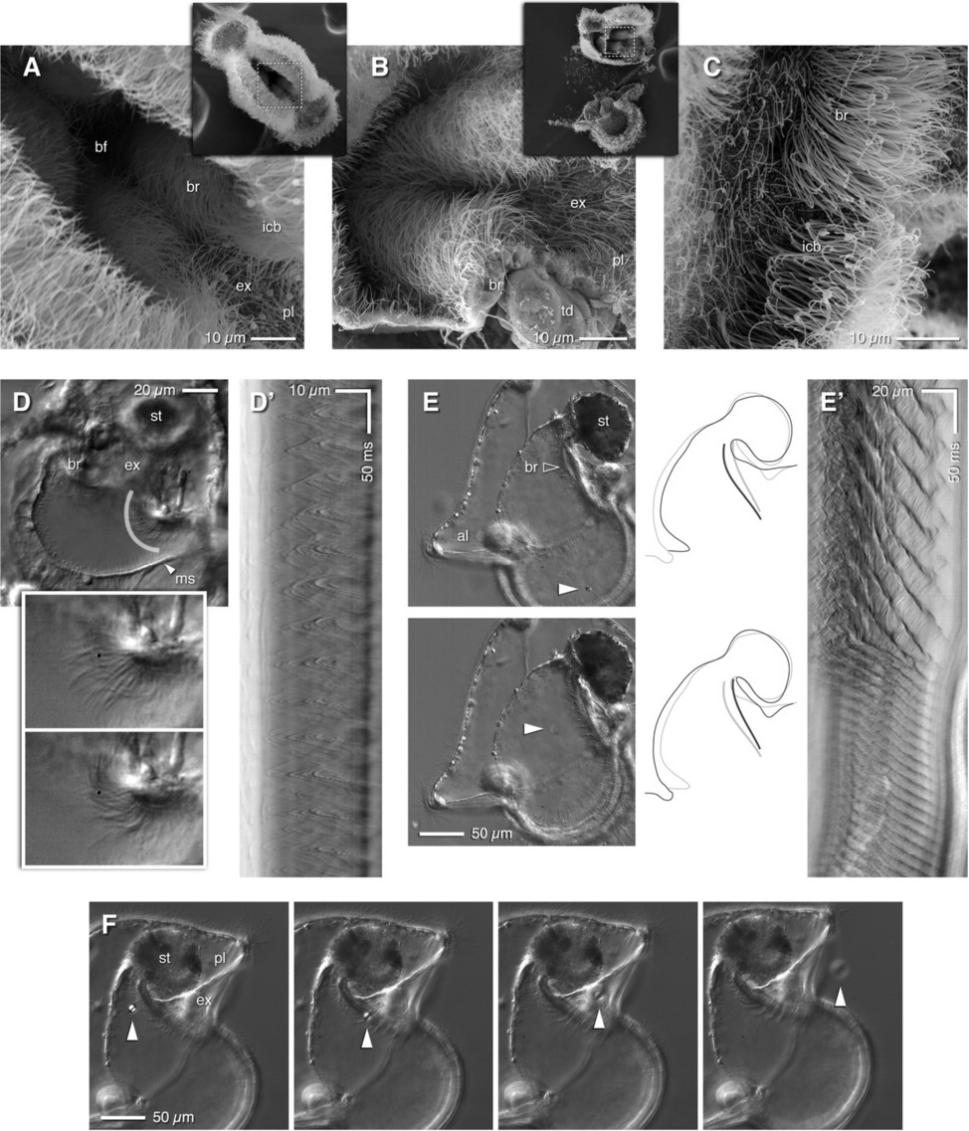
**Ciliated ridges in the buccal funnel. (A)** SEM of intact pilidium, viewed straight down the buccal funnel (bf). Inner ciliated band (icb) cilia point inward; buccal ridge (br) cilia point into exhalant gutter (ex) and outward, toward posterior lobe (pl). **(B)** Cracked pilidium exposing buccal ridges; right buccal ridge (br, lower one in figure) is cracked just above trunk disc (td). Cilia of both ridges point toward exhalant gutter (ex). **(C)** Pilidium cracked to expose the joint between inner ciliated band (icb) and buccal ridge (br). Cilia point in different directions, with a clear breakpoint. **(D)** View into the buccal funnel (Additional file 9: Video 9), showing beat orientation of buccal ridges (br) alongside exhalant gutter (ex); ms = circumesophageal muscle. Outset shows double-magnified frames at peak (forward stroke) and valley (just before recovery stroke). Kymograph (D’) of highlighted region shows that ciliary tips move backward almost as fast and far from the base as they do forward, suggesting the “resting” beat may achieve little net flow. **(E)** Pilidium trapped between slide and coverslip (Additional file 10: Video 10); although the lappets cannot flap well, at high density cells often blunder into the vestibule. This larva “notices” the cell (arrowhead), then accelerates buccal ridge (br) beat; this apparently initiates flow, drawing prey toward stomach (st) entrance. Tracings illustrate how the ridges are drawn backward, the funnel opens up, and the exhalant gutter clenches shut as the ciliary beat accelerates (top and bottom highlight before and after). Kymograph **(E’)** made just ahead of the buccal ridge indicates that the metachronal wave speeds up by ~3-fold. **(F)** Frame sequence in which a pilidium rejects a polystyrene bead (arrowhead); the object exits the buccal funnel through the buccal ridges, down the exhalant gutter (ex) below the stomach (st), and underneath the posterior lobe (pl).

Previous descriptions [26,29] suggest that the inner ciliated band continues up from the posterior margin of each lappet to the top of the esophageal funnel as ciliated or buccal ridges (“die Schlundfalten” in German literature), noted by early investigators [27,28,30]. The ciliated ridges within the esophageal funnel are clearly visible in live pilidia. However, two facts show that these buccal ridges represent distinct, tertiary ciliated bands: first, SEM examination of cracked-open larvae showed a distinct gap between the lappets’ inner band and the buccal ridges, a concomitant shift in the ridgeline, and a different phase of beat at time of fixation (Figure 5A, Figure 6A-C); second, live observation showed that the buccal ridges beat steadily at times when the inner ciliated band lies still. Video analysis and SEM examination concur that the buccal ridges beat toward a ciliated gutter that exits the esophageal funnel underneath the stomach – that is, between the trunk rudiments; toward the juvenile posterior (Figure 6D,F; Additional file 9: Video 9).

Notably, the buccal ciliated ridges do not beat vigorously at all times. In the absence of food, the buccal ridges exhibited a steady metachronal wave of low amplitude: most of the cilia did not appear to execute a complete power- and recovery-stroke cycle (Figure 6D; Additional file 9: Video 9, segment 1), often remaining bent in the direction of beat in both phases or curved very little for the recovery stroke (see kymograph in Figure 6D’). Thus it appears that the buccal ridges likely transport very little water in the absence of food. When food was captured, however, the beat of buccal ridge cilia increased by 3-5 fold; simultaneously, the ridges were drawn posteriorly and closer to the medial plane (Figure 6E; Additional file 9: Video 9, segment 2, and Additional file 10: Video 10). This appears to have the effect of dramatically increasing the draught through the funnel. The “activated” posture of the buccal ridges would seem to block the route to large objects (i.e., *Rhodomonas*) while transporting water out. We have not yet succeeded to document the flow pattern through the buccal chamber – pilidia proved averse to small tracers such as suspensions of small beads, bacteria, or milk – but we note that the switch to vigorous beating by the buccal ridges often coincides with the onset of an apparent upward draught that draws captured cells deeper into the funnel (see Additional file 10: Video 10). We cannot rule out other means that may transport cells up the buccal funnel walls to the stomach – e.g., handling by cilia along the funnel wall – when they are not near the buccal ridges.

### Cryptomonads try to escape the funnel

**Figure 7:**
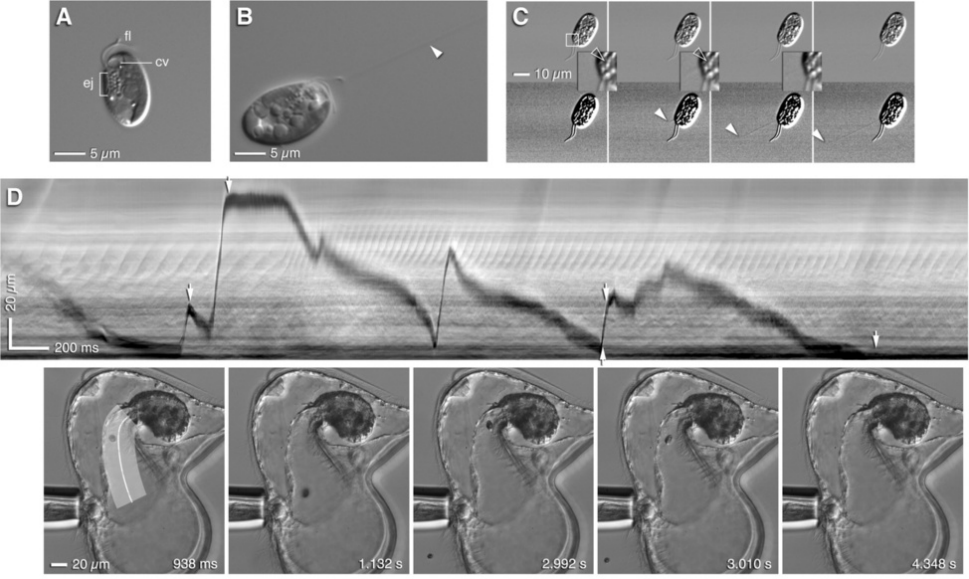
**Rhodomonas tries to escape the pilidium. (A)***Rhodomonas lens* (CCMP739) illustrating several rows of large ejectisomes (ej) lining the gullet from which the two flagella (fl) emerge; cv = contractile vacuole. **(B)***Rhodomonas* just after an abrupt jump; ejectisome filament(s) (arrowhead) remain along the path of the jump. **(C)** Another cryptomonad, *Storeatula* sp. (CCMP1868), trapped between slide and coverslip; four successive frames, 1 ms each, from high-speed video sequence in which a single ejectisome fired (because the cell was trapped, the filament extended away from the cell instead of making the cell move). Bottom row is contrast-enhanced to reveal the filament; arrowheads mark filament tip, which extends 26 μm in the time spanned by the two exposures (1.625 ms). Inset shows boxed region at 3x; hollow arrowhead points out the ejectisome which, because it changes from first to second frame, is apparently the one that fired. **(D)** High-speed video sequence of *M*. *alaskensis* pilidium maneuvering *Rhodomonas* up the buccal funnel (Additional file 11: Video 11); the kymograph was made by straightening the broad band indicated in the first of five frames shown below. Short arrows along the kymograph indicate the times of frames shown below. The dark streak is the path of the cell; the stomach entrance is at the bottom of the graph; thus the vertical distance represents distance from the stomach. Abrupt vertical excursions represent escape attempts, some of which correspond to multiple jumps in sequence. Most jumps are initiated when the cell is near the stomach entrance. Note the beat of the buccal ridges, which appears at middle height in the kymograph; a dramatic increase in beat frequency at ~1.5 s is associated with onset of a draught which sucks the cell back up the funnel.

We were initially puzzled by the motions of captured *Rhodomonas* as they approached the stomach entrance: cells appeared to be batted around within the funnel, at speeds as high as 1 cm/sec, as they bumped into the rapidly-beating buccal ridge cilia, or the dense ciliation near the stomach entrance. Yet this is physically impossible, because at a Reynolds’ number of <0.1 at most, the motion of *Rhodomonas* is nearly inertia-free. Hence we hypothesized that direct contact with dense, beating cilia must trigger some sort of escape response by these cells. We failed to detect any clear evidence of an escape swim, and in any case it is unlikely that flagellar motility could drive the cells at the observed speeds. However, cryptomonads possess special organelles, ejectisomes, which likely account for such rapid jumps. Both small and large ejectisomes have been described from a variety of species, and consist of a coiled ribbon which straightens rapidly to a long rod [31,32]. The large ejectisomes are apparent as one to four rows of beads along the gullet of the cell (Figure 7A).

We used high-speed video and DIC optics to confirm that *Rhodomonas lens* (and several other cryptomonads we culture as food for larvae) use the large ejectisomes to make rapid jumps of several body lengths (Figure 7B,C). This reaction could be stimulated by a variety of irritants, including pressure from a coverslip, added KCl, or the firing of a laser nearby. Therefore, we believe that this faculty accounts for the jumpiness of captured *Rhodomonas* as they approach the stomach. In our recordings *Rhodomonas* typically made fewer than a dozen individual long jumps before being swallowed (Figure 7D; Additional file 11: Video 11), which is approximately comparable to the number of ejectisomes visible in the typical cell. Also, *Rhodomonas* was essentially never able to actually escape the pilidium in our observations of two- to four-week-old larvae (although, see Additional file 12: Video 12): with jump lengths of 20-30 microns, it would have to have very good aim indeed to make it all the way – 200 microns or more – from the stomach entrance to freedom beyond the lappet margins.

## Discussion

### The pilidium larva is not like a trochophore

Our initial observations, by eye through a stereomicroscope, suggested to us the possibility that the pilidium uses its two lappets and their primary ciliated bands to achieve an opposed-band collection system, albeit one clearly only loosely analogous to that used by trochophores or veligers. In any case, this possibility is obviated by the finding that the lappets flick or flex open to grab a parcel of food-containing water as soon as cells of interest are detected at the lappet margin. Our finding that the inner ciliated band beats in the opposite direction to the primary band could also be suggestive, in that this pair could technically operate like the prototroch and metatroch (albeit without the intervening groove). Indeed, pending more detailed fate mapping studies, it could be that the primary ciliated band *is* homologous to the prototroch in some sense, although the metatrochs of feeding trochophores now appear to have evolved multiple times independently [33,34]. In any case, we found no evidence at all that these opposed bands are used analogously to the prototroch and metatroch in feeding trochophores. In the pilidium, the primary and inner ciliated bands do not even beat together: one or the other is normally active. In summary, we discovered no meaningful sense in which the pilidium’s feeding mechanism is like the trochophore’s.

The larval types with which the pilidium shares the most obvious structural or functional similarities are the bryozoan cyphonautes and the phoronid actinotroch. The cyphonautes uses a pair of ciliated ridges to drive a draught through a funnel at the top of which is a mouth and complete digestive system. However there are three sets of cilia on these ridges – lateral cilia produce the current, non-beating laterofrontal cilia act as sieves, and frontal cilia move particles to the mouth [35]. The actinotroch, like the pilidium, uses rapid movements of a muscular appendage (its hood) to gulp food: ciliated tentacles below the hood create a current, and when a particle contacts a tentacle the hood elevates to draw in the particle and surrounding water [11,12]. The similarities between the pilidium and these other larval forms are of course analogies, not homologies. The lack of obvious homologies here or with other feeding lophotrochozoan larvae (e.g. Müller’s larva, veligers) is consistent with our view that the pilidium, along with its feeding mechanism, is a novel invention of the nemerteans. Because of the long history of phylogenetic speculation involving larval forms, it is difficult to make comparative statements without unintentionally suggesting homology. Therefore, to avoid such entanglements, our further discussion will rely on analogies that are clearly free of phylogenetic implications.

### The pilidium is like a Venus’ fly trap

The feeding mechanism we have described is neither a passive sieve nor a purely local response; the whole animal responds to arrival of an interesting food item, and that response limits its ability to capture the next arrival. This is reminiscent of the Venus’ fly trap plant, which, when it closes upon an insect, must process that prey before restoring the trap. For the pilidium, the relevant delay is the time it takes to move captured cells from vestibule to buccal funnel. Some of our recordings (see segment 4 of Additional file 4: Video 4) show “greedy” larvae that apparently capture cells too quickly in succession, losing the first in gulping at the second. Because of the large variance in the time it takes to transport captured cells from vestibule to buccal funnel, only a very rough estimate of handling time is possible: granting that it is on the order of a second, this means the pilidium’s capture mechanism is probably saturated by prey densities greater than a few thousand per milliliter.

The collar cells embedded in the primary ciliated band are the likely detectors which, like trichomes of the Venus’ fly trap, may trigger the rapid entrapment of prey. Lacalli and West [26] described nerve fibers originating from monociliated collar cells in the primary ciliary band of the pilidium larva, which suggests that these cells are sensors of some sort; very recently, Hindinger et al. [36] showed that in wild-caught pilidia, presumed to be *Lineus albocinctus*, these collar cells (though apparently not the collar cells of the inner ciliated band or buccal ridges) are part of an FMRFamide-positive marginal nerve and nerve network spanning the lobes and lappets. Sensory cells with microvilli arranged in a circle around a cilium (i.e., stereocilia) have been described in epithelia of a wide variety of animal phyla, and they are typically interpreted as mechanoreceptors, although chemoreceptive or chemotactile function can not be excluded [37]. If collar cells in the ciliary bands of the pilidium are, indeed, mechanoreceptors, they are not trivial in nature. We have attempted to elicit a response by probing the larval margins with glass needles and balls of various sizes, and found them unmoved by such stimuli. We have tried repeatedly to feed pilidia polystyrene beads of the same size as *Rhodomonas*, and found that they eat them reluctantly if at all, and frequently reject them (sending them out the gutter at the back of the funnel) if they capture them by mistake. We found that pilidia *can* be fooled into capturing polystyrene beads if such beads have been properly seasoned, i.e. by soaking them overnight in a *Rhodomonas* culture.

Furthermore, although *Micrura alaskensis* pilidia readily eat all cryptomonads that we have offered them, including *Rhodomonas abbreviata*, *Chroomonas mesostigmatica*, and *Storeatula* sp. in addition to the standard fare, *Rhodomonas lens*, they are unenthusiastic at best toward non-cryptophyte flagellates such as *Dunaliella* or *Isochrysis*, and we have not succeeded in getting them to eat any kind of diatom or dinoflagellate. When pilidia do swallow green flagellates like *Dunaliella*, they appear to have difficulty digesting them, since intact cells accumulate in the stomach. These observations suggest some sort of selectivity, i.e. taste, because it is difficult to imagine how, within a millisecond, a pilidium could distinguish *Rhodomonas* from *Dunaliella* by touch alone. Nevertheless, the Venus’ flytrap also does not simply close up on any old glass bead, or rain drop, or stream of air [38] because the trichomes must be triggered in a timed sequence [39]. Perhaps the pilidium employs a similar trick.

### The pilidium is like a fluorescence-activated cell sorter

One of our most satisfying findings from high-speed video analysis is the remarkable control exhibited by pilidia over the deformations of the larval margin during food capture. This makes sense, immediately, of the extensive patterned musculature of the pilidial body. Coordinated movements of the lobes and lappets imply an integrated nervous control as well. By these devices, the pilidium captures cells not by handling them directly with cilia, so much as by re-directing streams of fluid to divert cells into the interior space, whence they can be handled more carefully by a fluid draught. This is analogous to the design of an automated cytometer like a FACS, which hydrodynamically focuses a stream carrying individual cells, runs them past a detector, and diverts the stream according to the readout, without handling the cells directly until they reach their destination. Based on our observations of the cryptomonad escape attempts, we suspect that the pilidium uses this strategy to capture cells without eliciting their defensive responses. We rarely saw *Rhodomonas* attempt an escape until it approached the buccal ciliated ridges or the stomach entrance, both points at which the cell is finally contacted directly by dense ciliary fields. Thus it seems that the pilidium contrives to use fluid streams, in contrast to direct manipulation or ciliary sieving used by other ciliary suspension feeders, to get captured prey deep enough into the interior space that by the time the prey notices it’s been caught, it’s too late.

This interpretation implicitly supposes a target prey item which has the kind of escape response that cryptomonads possess, and in turn implies that the larval form might represent an adaptation to such traits. Cultured cryptomonads are especially good food for many invertebrate larvae – the widespread use of *Rhodomonas lens* by larval biologists as laboratory food attests to this fact – and may be so in nature too [40,41]. This is perhaps not only because these flagellates are vacuole-free, naked cells: they also retain red-algal-derived light-harvesting proteins that help them to photosynthesize in dim light, possibly allowing them access to nutrients that are otherwise limiting in the photic zone [42]. Many invertebrate larvae, when caught in plankton tows, have stomachs colored with the rich red, blue-green, or ochre colors that, in the lab, reflect a diet of *Rhodomonas* or other cryptophyte. Furthermore, a wide diversity of phytoplanktiverous invertebrate larvae have a large vestibule for a mouth: in addition to the pilidium this set includes all of the echinoderm dipleurulas, phoronid actinotrochs, the mitraria of *Owenia*, the bryozoan cyphonautes, and brachiopod glottidias. Cryptomonads likely appeared on Earth well before most of these larval types evolved [43,44]. It is not inconceivable that convergences in form and feeding mechanism among animal larvae might be driven by a shared appetite for this one especially good planktonic food, just as grasses have shaped the convergent evolution of diverse terrestrial herbivores.

### The pilidium is like a baleen whale

The pilidium larva engulfs a substantial volume of water with its prey, which water must then be separated from the prey and ejected from the body without liberating the captured food item. Filter-feeding whales do this by squeezing the engulfed water through baleen [45]; the inner ciliated bands along the lappets of the pilidium and the buccal ridges appear to serve a similar function, in that they both beat outward (thus presumably removing water) and also erect a transient fence across the escape route. It is not clear to what extent the pilidial larval body can actually squeeze against the captured water: the esophageal funnel is equipped with circumferential muscles and can also flatten either mediolaterally or frontally, but it does not immediately seem likely that the lappet epithelium and associated musculature can pressurize the ball of water held within the vestibular space. Instead we suspect that the separation of water from food is accomplished primarily by ciliary action of the inner lappet band and the buccal ridges. It is perhaps worth noting that, based on observed deformations of the lappets, the pilidium engulfs on the order of a nanoliter of water with each gulp, from which a single cell – about a picoliter – is eventually swallowed. Surely this feat of 1000-fold concentration of the plankton, accomplished within mere seconds, is on par with the exertions of any whale.

We have tried to directly visualize flow within the funnel using various inert particles, but have not yet succeeded, largely because 1) the internal ciliary band system is more or less at rest unless it has captured a food particle, and 2) pilidia detect and exhibit aversion to the tracers we have tried. Until we can directly probe flow within the esophageal funnel, we surmise from the behavior of cells that: the ciliary bands create a circulation that enters between the lappets and exits the gutter at the back of the funnel; fluid shear alongside this draught carries captured cells upward; large objects like *Rhodomonas* can’t pass the dense, beating cilia of the buccal ridges, and are hence pushed up the narrowing funnel to the point where they can be directly handled by the dense ciliary field at the stomach entrance. It is at this point – as much as a second or three after initial engulfment – that *Rhodomonas* detects its circumstances and makes its repeated, futile attempts to escape.

### Growth and change in feeding modes during development and evolution

The mechanism we describe here typifies “mature” larvae of *M*. *alaskensis*, that is, ones that will still grow and change, but whose feeding apparatus and capture repertoire is complete. The basic outlines are likely to apply to all conventional (hat-like) pilidium larvae; we have observed similar behavior in the larvae of *Cerebratulus marginatus*, *Lineus flavescens*, and an undescribed lineiform species collected locally. Young pilidia do not exhibit the full repertoire. At onset of feeding (~ 3 days in *M*. *alaskensis*), lappets do not extend far beyond the funnel margin yet, and cannot be flapped. Even at the two-pairs-of-discs stage (~2 weeks), *M*. *alaskensis* pilidia have not yet achieved fine control over the deformations of the anterior and posterior lobe margins, although they can move the lappets quite well at this stage. Furthermore, we found that pilidia can feed even after both lappets or the anterior lobe are amputated (we have not measured the relative efficiency of operated pilidia). Therefore, not all of the techniques documented here are essential for feeding.

Further study of two unusual types of pilidium, belonging to species presently thought to branch basally within the Pilidiophora, may illuminate the origins or adaptability of the pilidial feeding mechanism. *Pilidium auriculatum* is the larva of *Hubrechtella*[21,46], a representative of the one non-heteronemertean family within the Pilidiophora [14], and instead of lappets has, in the equivalent position, two narrow straps. The musculature of *pilidium auriculatum* seems much less well developed than that of conventional pilidia, and when we have observed it feeding on *Rhodomonas* we saw no evidence of the flicks and snatches that typify conventional pilidia (GvD unpublished data). Another unusual form, *pilidium recurvatum*, recently matched to a basal heteronemertean *Riserius*, feeds in an uncharacterized manner on an unknown food source, likely using an extended trumpet-shaped oral funnel with complex internal ciliation, but no apparent ciliated bands and nothing analogous to the lobe and lappet musculature of conventional pilidia [47]. Whether these represent derived, simplified types or something like ancestral forms is (and may always remain) unclear, but in either case it would be valuable to know how (and on what food) these variants make a living, and how well they perform compared to the canonical type of pilidium.

## Conclusions

All pilidia have in common that the juvenile develops from imaginal discs (ref. [21], and references therein). All feeding forms also share the inflated blastocoel and funnel-like mouth, traits which are unknown amongst the assortment of macrophagous or non-feeding planuliform larvae of palaeo- and hoplonemerteans. It is inescapable that the pilidium is not just a highly-derived version of some familiar larval form, but qualifies as a singular invention of a new larval body plan. By extension, the various similarities between the pilidium and other planktotrophic larvae – inflated blastocoel, ciliary band organization, funnel-like mouth, or even pouches of proliferative cells set aside to make the juvenile – result from convergence upon functional solutions to shared demands.

## Materials and methods

### Animals, larvae, and their food

Adults of *Micrura alaskensis* were obtained by shovel from intertidal sand flats around Charleston, OR. Gravid adults were minced to release gametes; oocytes were inseminated with dilute sperm, then larvae were raised in glass gallon jars of filtered natural seawater with regular water changes and feeding on *Rhodomonas lens* (CCMP739), as described by Maslakova [18]. *Rhodomonas* was raised on either natural light or constant light in f/2 medium. For feeding experiments we found it necessary to use only algal cultures in growth phase; dense, aging cultures, even if seemingly composed of viable cells, did not inspire great appetites in tethered pilidia.

In addition to pilidia of *M*. *alaskensis* we used a wild-caught pilidium likely belonging to *Lineus flavescens*, or a closely related species, to illustrate the non-beating cilia of putative sensory cells along the margins of pilidial larval lappets and lobes. This pilidium was among many collected in plankton samples taken off a dock in Charleston, OR in January 2013.

### Fixation, staining, and confocal microscopy

Larvae were relaxed 5-10 min in a 50:50 mix of filtered seawater and 0.35 M MgCl_2_, fixed for 1 h in 4% paraformaldehyde (Electron Microscopy Sciences) in filtered seawater, rinsed thoroughly in Phosphate Buffered Saline (PBS) and permeabilized with PBT (PBS + 0.1% Triton X-100), then stained with 1 U/200 μl Bodipy FL phallacidin (Invitrogen) in PBT. After rinsing, larvae were attached to poly-lysine coated coverslips and mounted in Vectashield (Vector Laboratories). Specimens were examined on an Olympus FluoView 1000 or Biorad Radiance 2000 LSCM using a Plan Fluor 40x 1.3 NA oil lens. Image stacks were projected and adjusted using ImageJ.

### Scanning electron microscopy

Larvae were relaxed 5-10 min in a 50:50 mix of filtered seawater and 0.35 M MgCl_2_, then killed by adding a drop of 1% formalin to sea water with larvae, and fixed by first replacing this mixture with a volume of 2.5% glutaraldehyde in 0.2 M Millonig’s Phosphate Buffer, pH 7.4 (Electron Microscopy Sciences), and after a few minutes, adding an equal volume of 4% OsO4 (Electron Microscopy Sciences) to a final concentration of 1.25% glutaraldehyde and 2% OsO_4_. Fixed larvae were rinsed thoroughly in distilled water after 1-2h, then dehydrated through a series 30%-50%-70% EtOH for storage. For examination, larvae were dehydrated to 100% EtOH (through a series 80%-90%-95%-100%-100%-100%), dried using a CO_2_ EMS K850 Critical Point Drier, then sputter-coated with gold using an Emscope SC500. Specimens were examined using a Tescan Vega II SEM.

### Video microscopy

Larvae were held by capillary suction on the end of a microcapillary pipette, shaped and fire-polished with a Narishige MF-9 microforge. For low-power recordings, larvae were held in a dish or drop slide on the stage of a Leica MZ10FL stereomicroscope and filmed with either a Sony DXC33 color CCD camera (real-time recordings) or a Photron FastCam MC1 CMOS camera (high-speed recordings, 500 or 1000 frames per second). Higher-power recordings used an Olympus BX51 DIC microscope with Plan Fluor objectives, and a chamber made out of a drop slide and a coverslip supported by cut pieces of glass capillary (1.2 mm OD) held in place by vacuum grease. Larvae held in such a chamber were approximately 1 mm from any wall. Some recordings involved larvae trapped between a slide and coverslip supported by clay feet. For still images we used a Point Grey Research Grasshopper 2 monochrome CCD camera. All image analysis was conducted using ImageJ.

## Competing interests

The authors declare that they have no competing interests.

## Authors’ contributions

SvM raised the larvae; GvD and RBE did the high-speed video and analysis thereof; SvM and GvD did the confocal and scanning electron microscopy; GvD made the figures and drafted the manuscript; all three authors jointly conceived of the study and developed the interpretations presented here. All authors read and approved the final manuscript.

## Supplementary Material

Additional file 1: Video 1 Essential features of the external ciliation and beat in the pilidium of *M*. *alaskensis* (corresponds to Figure 1B). Recorded at 500 fps / playback at 30 fps = ~16x slow-motion. Click here for file

Additional file 2: Video 2 Frontal view of trapped, feeding pilidium of *M*. *alaskensis*, real time, showing two captures and more attempts (corresponds to Figure 1F). Click here for file

Additional file 3: Video 3 Side view of trapped, feeding pilidium of *M*. *alakensis*, real time (corresponds to Figure 1G). Click here for file

Additional file 4: Video 4 Excerpts of six low-magnification high-speed sequences of *M*. *alaskensis* pilidia feeding while held by a capillary pipet (corresponds to Figure 2). All were recorded at 500 fps / 30 fps playback = ~16x slow-motion. Click here for file

Additional file 5: Video 5 High-speed video sequence illustrating the relationship between the path of a captured *Rhodomonas* to lappet movement and the non-beating cilia in the primary ciliated band of *M*. *alaskensis* (corresponds to Figure 4D). Recorded at 500 fps / playback at 30 fps = ~16x slow-motion. Click here for file

Additional file 6: Video 6 This high-speed video sequence illustrates the outward beat of the inner ciliated band of a pilidium of *M*. *alaskensis* upon particle capture (corresponds to Figure 5B,C). Recorded at 500 fps / playback at 30 fps = ~16x slow-motion. Click here for file

Additional file 7: Video 7 High-speed video sequence depicting erection of the inner ciliated band in a feeding pilidium of *M*. *alaskensis*, apparently creating a fence across the opening to the outside world (corresponds to Figure 5D). Recorded at 500 fps / playback at 30 fps = ~16x slow-motion. Click here for file

Additional file 8: Video 8 Two excerpts from high-speed sequences of a pilidium of *M*. *alaskensis* in which the captured *Rhodomonas* passes over the lappet while remaining nearly in focus (no corresponding figure). In each case the inner ciliated band erects on the opposite side and elsewhere around the lappet margin, but remains more or less at rest in the region over which the captured cell entered. Recorded at 500 fps / playback at 30 fps = ~16x slow-motion. Click here for file

Additional file 9: Video 9 Down-the-funnel view of a trapped pilidium of *M*. *alaskensis* showing motion of the buccal ridge cilia “at rest”, i.e., not beating as fast as when food is present (corresponds to Figure 6D). The second segment of the video shows (at a lower magnification) a capture event during which the ciliary beat of the buccal ridges speeds up dramatically. Recorded at 500 fps / playback at 30 fps = ~16x slow-motion. Click here for file

Additional file 10: Video 10 High-speed video of a pilidium *of M*. *alaskensis* trapped between slide and coverslip (corresponds to Figure 6E and 6E’) showing a change in ciliary beat of the buccal ridges after capture of *Rhodomonas*. Recorded at 500 fps / playback at 30 fps = ~16x slow-motion. Click here for file

Additional file 11: Video 11 This high-speed sequence features a pilidium of *M*. *alaskensis* which transports a captured cell toward the stomach despite repeated attempts by *Rhodomonas* to escape (corresponds to Figure 7D). The depleted cell is finally swallowed by the pilidium. Recorded at 500 fps / playback at 30 fps = ~16x slow-motion. Click here for file

Additional file 12: Video 12 extended clip from one of the components of Additional file 4: Video 4 (corresponds to Figure 2F). Remarkably, in this one instance the captured cell escaped the buccal funnel by jumping between the buccal ridges, only to be recaptured immediately. Recorded at 500 fps / playback at 30 fps = ~16x slow-motion. Click here for file
